# Case Report: Diverse pediatric phenotypes of *RELA* frameshift variants: comparison of two cases

**DOI:** 10.3389/fimmu.2026.1756745

**Published:** 2026-02-18

**Authors:** Ling Hou, Lu Yin, Chengguang Zhao, Yue Du

**Affiliations:** Department of Pediatrics, Shengjing Hospital of China Medical University, Shenyang, China

**Keywords:** autoinflammation, Behçet’s disease, NF-κB, pediatrics, *RELA*

## Abstract

**Background:**

Variants in *RELA* (which encodes the p65 subunit of NF-κB) can cause a monogenic autoinflammatory disease with clinical manifestations that range from mucocutaneous lesions (Behçet’s disease-like) to systemic inflammation. However, the diversity of the phenotype and its penetrance are uncertain.

**Case presentation:**

Patient 1 (p.Asp465Thrfs14) presented with classic Behçet’s disease-like symptoms of oral and genital ulcers, fever, and elevated inflammatory markers. Colchicine (0.25 mg once daily) with low-dose glucocorticoids led to remission. Exploratory *in vitro* assays using murine fibroblasts indicated increased TNF-α–induced apoptosis associated with the *RELA* variant. Patient 2 (p.Glu495Serfs6) had no mucosal lesions but experienced recurrent high fever, hyperferritinemia, uveitis/scleritis, and progressive bilateral sensorineural hearing loss. Because IL-1 blockers were not available, we administered adalimumab as a steroid-sparing treatment. Defervescence was achieved within 6 months of adalimumab therapy, with stabilization of inflammatory markers and hearing thresholds during the entire 9-month follow-up. PBMCs from Patient 2 and the carrier mother that were stimulated by LPS had decreased induction of two genes targeted by NF-κB (*BCL2A1*, *TRAF1*), and the proband (but not the mother) had markedly increased IL-6 secretion.

**Conclusions:**

C-terminal truncations in the transcriptional activation domain of *RELA* lead to haploinsufficiency and an inflammatory phenotype that depends on the cell type and stimulus. *RELA* screening should be considered in the evaluation of children with unexplained autoinflammatory presentation, even in the absence of mucosal ulceration.

## Introduction

Germline defects that affect the canonical NF-κB pathway can cause pediatric autoinflammation with features similar to Behçet’s disease (BD). In addition to the genes responsible for polygenic risk of BD (e.g., *HLA-B51*, *ERAP1*, *IL-10*/*IL-23R*), monogenic inborn errors that affect NF-κB signaling, including certain variants of *RELA* (which encodes the p65 subunit of NF-κB), account BD-like symptoms in a subset of patients, but these patients have an earlier onset and variable extra-mucosal involvement (1, 2). Previous studies (3, 4) have shown that pathogenic *RELA* variants were associated with *RELA*-associated inflammatory disease (RAID), a spectrum of disorders characterized by mucocutaneous inflammation and systemic immune dysregulation. Most reported variants affected the C-terminal transactivation domain (TAD) of *RELA* and acted through loss-of-function (LoF) or dominant-negative (DN) mechanisms.

A 2017 study first described human *RELA* haploinsufficiency as a cause of chronic mucocutaneous ulceration with TNF-driven apoptosis and salutary responses to anti-TNF therapy. Since then, the phenotypic spectrum of this disease has expanded markedly (3). In particular, subsequent series reported familial BD-like ulceration and neuromyelitis optica from a C-terminal truncation in the *RELA* gene (p.His487Thrfs*7) and several families with incomplete penetrance and immune dysregulation. These recent studies indicate that RAID is a clinically heterogeneous entity (4, 5). Other research reported that *RELA* haploinsufficiency led to a condition similar to autoimmune lymphoproliferation syndrome (ALPS) with autoimmune cytopenias, thus broadening the immune phenotype beyond the mucosa (6).

A recent study found that DN *RELA* variants were present in patients who had a type I interferonopathy with autoinflammation/autoimmunity due to enhanced TLR7-MYD88 signaling and increased production of IFN-I/III, and that the mechanism was distinct from dosage reduction but there was overlap in the clinical presentation (7). These advances motivated early genetic evaluation of children who had unexplained autoinflammation — even without orogenital ulcers — and led to a pathway-guided approach to therapy (8).

## Case presentation

### Patient 1

A 12-year-old Han Chinese girl presented in October 2024 with an 8-year history of recurrent and painful oral aphthae, a 4-month history of vulvar ulcers that tended to flare after menses, an intermittent high-grade fever up to 39.5°C, and occasional arthralgia. On admission, she initially appeared well, but an examination showed multiple aphthous scars on the gingiva/buccal mucosa and several tender superficial ulcers on the labia minora. The laboratory tests indicated high levels of acute-phase reactants (CRP: 48mg/L, ESR: 62mm/h), negative autoimmune serology tests (ANA, rheumatoid factor, and HLA-B51) and unremarkable results from a chest CT and abdominal ultrasound. The working diagnosis was BD-like condition pending further evaluation (**Supplementary Table 1**).

**Figure 1:**
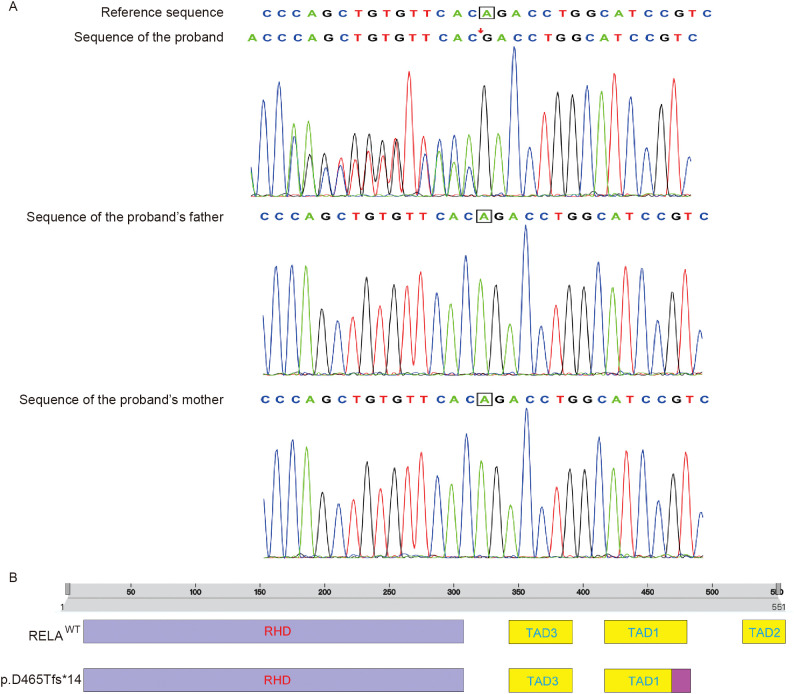
Trio-based whole-exome sequencing (WES) (Patient 1). **(A)** A heterozygous *de novo* frameshift mutation in *RELA*, c.1392delA (p.Asp465Thrfs*14), in the proband but not in the mother or father, identified by trio-based WES and confirmed by Sanger sequencing. **(B)** Mapping of the *RELA* p.Asp465Thrfs*14 frameshift variant to the C-terminal transactivation domain (TAD) of *p65* (*RELA*) and prediction of defective transactivation.

Whole-exome sequencing identified a heterozygous *de novo* frameshift mutation in *RELA*: c.1392delA (p.Asp465Thrfs*14). Protein-domain mapping (**Figure 1**) indicated this lesion was in the C-terminal TAD of the p65 subunit of *NF-κB* (*RELA*), downstream of the Rel-homology DNA-binding domain. This led to a predicted truncation of p65 and impaired transcription of *NF-κB* (**Supplementary Table 2**). Both parents tested negative for this variant, indicating this was a *de novo* mutation. These results are concordant with previously described *RELA* LoF variants in the TAD that destabilize p65, reduce p65 phosphorylation, and decrease the expression of genes induced by NF-κB (5).

Patient 1 was treated with low-dose colchicine (0.25 mg once daily) combined with a short course of systemic corticosteroids (30 mg qd, tapered and discontinued after 2 months). This treatment led to rapid resolution of fever and mucocutaneous ulcerations, with sustained clinical remission during follow-up. This *de novo RELA* variant, together with the rapid response to treatment and the absence of BD-associated HLA-B51, supports classification within the RAID spectrum rather than polygenic BD.

**Figure 2:**
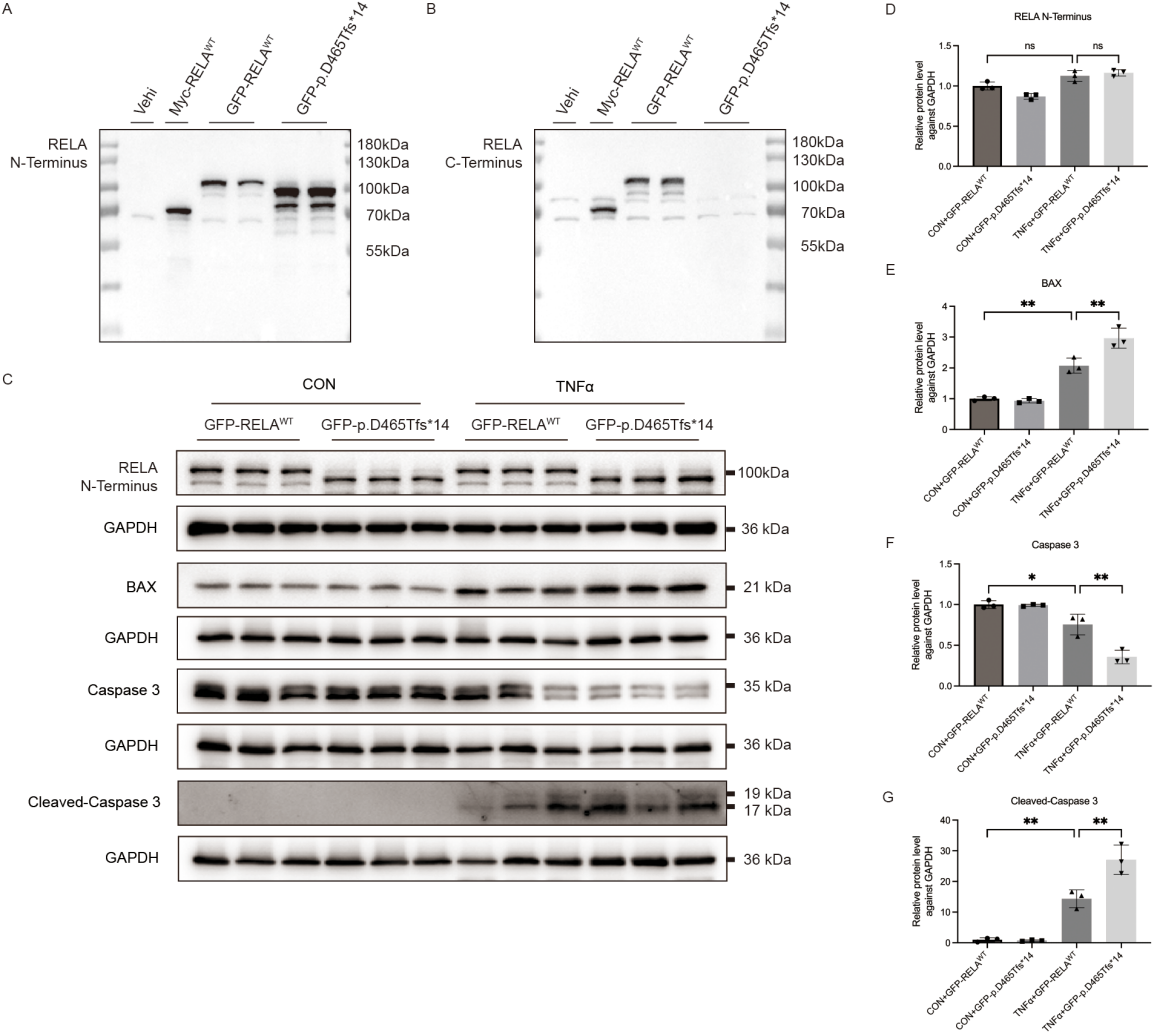
Effect of transfection of murine fibroblasts (L929) with wild-type *RELA* or a *RELA* frameshift variant (p.Asp465Thrfs*14), followed by TNF-α challenge (10 ng/mL), on the expression of apoptosis markers (Patient 1). **(A)** Western blotting using an N-terminal RELA antibody to assess protein expression in murine fibroblasts transfected with different plasmids. **(B)** Western blotting using a C-terminal RELA antibody to assess protein expression in murine fibroblasts transfected with different plasmids. **(C)** Western blotting of apoptosis-related proteins (BAX, Caspase-3, and cleaved Caspase-3) in transfected fibroblasts following TNF-α stimulation. **(D–G)** Quantification of the results in C.

To assess the pathogenic plausibility of this classification, we performed exploratory *in vitro* assays using murine fibroblasts that were transiently transfected with plasmids encoding wild-type *RELA* or the patient’s p.Asp465Thrfs*14 variant. The results showed that TNF-α stimulation (10 ng/mL) led to higher levels of two apoptosis markers (BAX and Cleaved-Caspase 3) in cells with the frameshift variant (**Figure 2**). Although this experiment was performed *in vitro* with mouse cells that over-expressed the target gene, the results are consistent with diagnosis of a *RELA*-deficient state, namely, TNF-driven apoptosis with impaired induction of NF-κB-dependent anti-apoptotic signaling in non-hematopoietic cells (1, 5, 8). This mechanism appears to be responsible for mucosal ulceration in RAID and related disruption of the NF-κB pathway.

In summary, our integration of data on the clinical phenotype, genotype, structural mapping, and supportive functional experiments implicate a truncation of the TAD of *RELA* as the cause of a pediatric BD-like mucocutaneous syndrome. Importantly, the patient had an excellent and rapid response to colchicine plus low-dose steroids. The *in vitro* alteration of apoptosis is consistent with recent models in which reduced production of p65 increases TNF-α-mediated cytotoxicity at mucosal surfaces (3). This proposed mechanism connects this specific gene variant to the patient’s disease and provides a rationale for therapy that targets the NF-κB pathway.

### Patient 2

A 10-year-old boy presented in January 2025 with a 1-year history of recurrent high-grade fever (peaking at 40°C), marked hyperinflammation (CRP>200mg/L; ferritin:3,500ng/mL), and progressive bilateral sensorineural hearing loss (SNHL). He had no oral or genital ulcers and no cutaneous lesions. The results from tests for infection and autoimmunity were unremarkable. On admission, the laboratory tests showed neutrophilic leukocytosis (WBC: 23.48×10^9^/L; neutrophils 78.6%). A chest CT was normal, but an abdominal CT demonstrated mesenteric lymphadenopathy with reduced hepatic attenuation. A previous ophthalmology examination documented uveitis/scleritis that was responsive to corticosteroids. We initiated empiric therapy consisting of ceftriaxone-tazobactam (2.0 g qd for 1 week) and low-dose systemic corticosteroids (40 mg qd),and this was followed by transient decreases in the levels of CRP and ferritin. An audiometry examination confirmed moderate-to-severe bilateral SNHL, and suggested a systemic autoinflammatory process rather than a classical autoimmune disorder (**Supplementary Table 1**).

**Figure 3:**
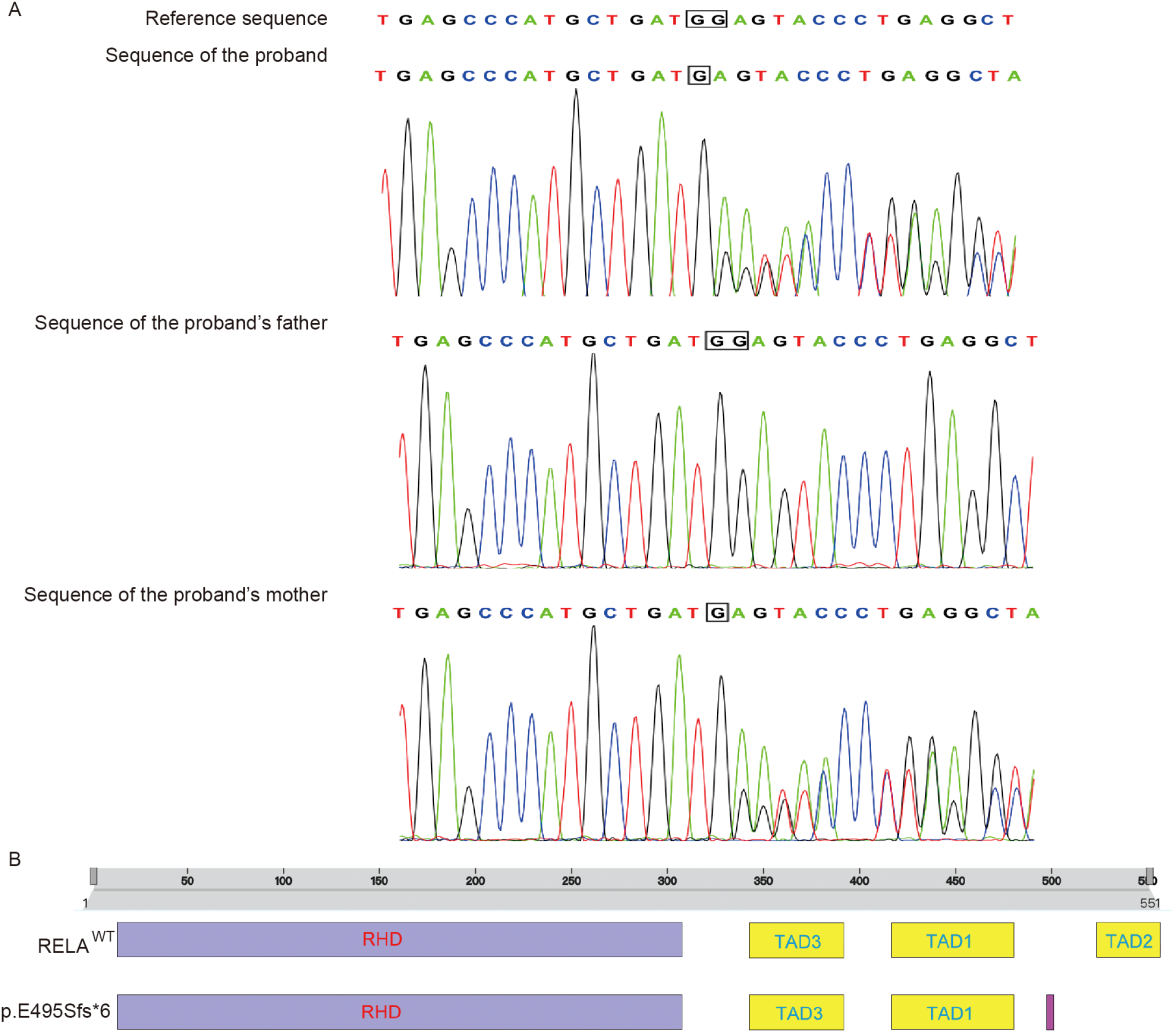
Trio-based whole-exome sequencing (WES) (Patient 2). **(A)** A heterozygous frameshift mutation of *RELA* in the proband and the mother, c.1483delG (p.Glu495Serfs*6), identified by trio-based WES and confirmed by Sanger sequencing. **(B)** Mapping of the *RELA* c.1483delG (p.Glu495Serfs*6) variant to the C-terminal transactivation domain (TAD) of *p65* (*RELA*) and prediction of defective transactivation based on TAD-truncating variants in other families with RAID.

Whole-exome sequencing identified a heterozygous *RELA* frameshift mutation, c.1483delG (p.Glu495Serfs*6). Trio-based whole-exome sequencing demonstrated that this variant was inherited from the mother (**Figure 3**). This mutation is in the C-terminal TAD of *p65*, consistent with LoF and attenuated signaling by the canonical NF-κB pathway, showing a pattern of NF-κB activity similar to C-terminal truncations in other cohorts with a BD-like condition (e.g., p.His487Thrfs*7) (**Supplementary Table 2**) (4, 5).

**Figure 4:**
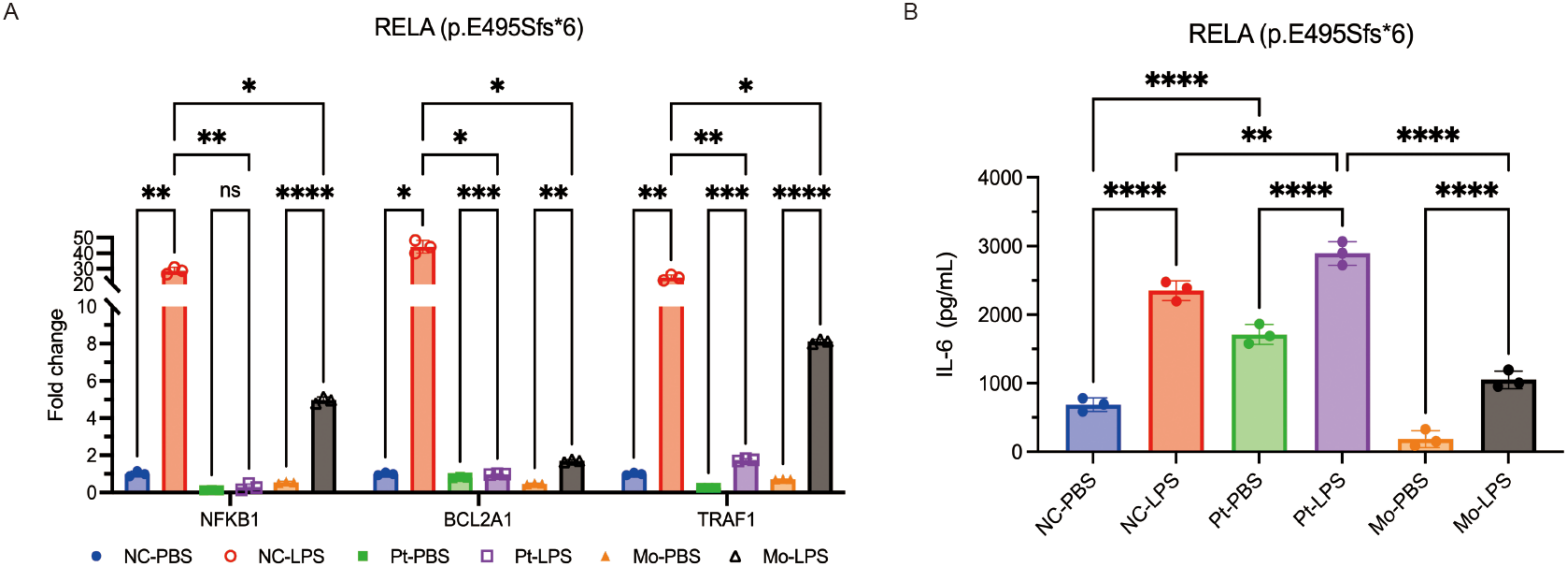
*Ex vivo* stimulation of PBMCs by LPS (Patient 2). **(A)** qPCR (fold-change in expression relative to an unstimulated control) showed that LPS stimulation (5 μg/mL, 24 h) led to impaired induction of *BCL2A1* and *TRAF1* in the patient (Pt) and the carrier mother (Mo) relative to the normal control (NC, from a healthy donor), indicating defective *RELA* transactivation. **(B)** ELISA indicated elevated IL-6 in the patient but not in the mother relative to the control, highlighting variable expressivity and stimulus-dependent cytokine dysregulation. Data are presented as fold-change relative to unstimulated controls.

To assess the consequences of this mutation, PBMCs from the proband, the carrier mother, and a healthy control were cultured and stimulated with lipopolysaccharide (LPS, 5 µg/mL) for 24 h; unstimulated cells were used as a control. qPCR showed impaired induction of the NF-κB target genes, *BCL2A1* and *TRAF1*, in both the proband and the carrier mother, expressed as fold-change relative to the unstimulated control (**Figure 4A**). Consistently, ELISA of culture supernatants demonstrated markedly increased IL-6 secretion in the proband but not in the mother (**Figure 4B**), highlighting variable expressivity. These findings suggest that the same transactivation defect led to different cytokine responses. This result is congruent with prior research of *RELA* kindreds which showed reduced p65/p-p65 response due to cell-specific and stimulus-specific hyper-secretion of IL-6/TNF in some individuals. Thus, incomplete penetrance can explain the different symptoms in the boy and his mother (3, 5).

Because IL-1 receptor blockade has only recently become available in mainland China and lacks pediatric indications, we administered adalimumab (an anti-TNF monoclonal antibody) as a steroid-sparing strategy. After initiation of this therapy, the patient achieved defervescence within 6 months. During the 9-month follow-up, the inflammatory markers remained stable and there was no further deterioration in hearing. There were no new mucosal symptoms or infectious complications. The success of this treatment is consistent with previous reports that TNF inhibition can be effective for patients with *RELA*-associated and BD-like disease, and also reinforces the heterogeneity and variable penetrance that occurs in families with the same *RELA* variant (4, 8).

Taken together, the TAD-truncating frameshift mutation in *RELA*, the failed transactivation in PBMCs, and the selective hyper-secretion of IL-6 suggest the symptoms of this patient were due to a loss of canonical NF-κB signaling with stimulus-dependent cytokine skewing. This is consistent with earlier studies of *RELA* which showed that TNF-driven cytotoxicity occurs when NF-κB-dependent pro-survival signaling is blunted, and with studies of larger familial cohorts which reported a spectrum of immune-dysregulation (mucocutaneous ulcers ± irritable bowel disease [IBD], ocular involvement, and variable cytokine responses). These data suggest that *RELA* testing should be performed in children with periodic fever and sensory-organ inflammation, even without mucosal ulcers, and provide a rationale for anti-TNF therapy when IL-1 blockade is not feasible (1, 3, 5).

## Methods

### Ethical approval

This study was approved by the Ethics Committee of Shengjing Hospital (approval No. 2025PS1470K). Written informed consent for participation, genetic testing, and publication of clinical and genetic data was obtained from the parents or legal guardians of both patients, in accordance with the Declaration of Helsinki.

### Genetic analysis

Trio-based whole-exome sequencing (WES) was performed for both patients and their parents using a clinical diagnostic pipeline. Exonic regions were captured using a commercial exome enrichment kit (Tiangen) and sequenced on a high-throughput platform. The mean sequencing depth exceeded 100× and more than 99% of targeted bases were covered at 20× or more, including the *RELA* locus. Sequence alignment, variant calling, and annotation were performed using standard bioinformatic pipelines. Variants were interpreted according to the American College of Medical Genetics and Genomics (ACMG) guidelines. The identified *RELA* variants were confirmed by Sanger sequencing. Parental samples were analyzed concurrently to determine inheritance. No other pathogenic or likely pathogenic variants were identified in other genes previously associated with BD or related autoinflammatory conditions, based on trio-based whole-exome sequencing and standard criteria for variant interpretation.

### *Ex vivo* functional assays

Peripheral blood mononuclear cells (PBMCs) were isolated from the proband, the carrier mother, and a healthy control by density-gradient centrifugation. PBMCs were stimulated with lipopolysaccharide (LPS, 5 µg/mL) for 24 h, and unstimulated cells were used as a control. Total RNA was extracted and reverse-transcribed, and quantitative PCR was performed to assess expression of NF-κB -dependent target genes (*BCL2A1* and *TRAF1*). Gene expression was normalized to *ACTB* (beta-actin) using the ΔΔCt method. Culture supernatant levels of IL-6 were measured using a commercial ELISA kit (Elabscience, E-HSEL-H0003) according to the manufacturer’s instructions. All samples were assayed with technical replicates. Given the limited number of available samples, these functional assays were primarily descriptive and were performed for mechanistic support rather than formal statistical inference.

### Statistical analysis

Data are presented as mean ± standard deviation. Given the exploratory and descriptive nature of the functional experiments and the limited sample size, no formal statistical comparisons were performed. Gene expression and cytokine levels are presented as fold-change relative to unstimulated controls.

## Discussion

We identified two patients with C-terminal frameshift mutations in *RELA* (p.Asp465Thrfs*14 in Patient1; p.Glu495Serfs*6 in Patient2). The key clinical and immunological differences of the two patients, including mucosal-dominant disease versus SNHL/uveitis, cytokine profiles, and therapeutic responses, are summarized in **Supplementary Table 1**. Each variant led to truncation of the *p65* TAD and failure of NF-κB-dependent transcription of pro-survival genes. This LoF, first established in human *RELA* haploinsufficiency and corroborated in cellular and animal models, explains why tissues that rely on rapid NF-κB-mediated protection (e.g., mucosa and stromal compartments) are vulnerable to TNF-mediated apoptosis. Our results also provide a resolution to the paradox of high-grade inflammation coexisting with impaired canonical NF-κB signaling (3).

TAD-truncating variants — including the two frameshift mutations described here — decrease the transactivation of *RELA* and the induction of anti-apoptotic proteins (BCL2A1 and TRAF1). This sensitizes stromal and epithelial cells to TNF-induced cytotoxicity, and thus explains mucosal ulceration in RAID. In Patient 1, we assessed apoptosis in murine fibroblasts following TNF-α challenge (**Figure 2**). Our results align with familial and cohort studies in which *RELA* truncations increased caspase activation and decreased NF-κB reporter activity following stimulation by TNF (3–5).

Importantly, not all *RELA* variants have the same effect. A recent study defined DN mutant proteins that dimerize with WT p65/p50 and drive upregulation of type-I interferon (IFN-I) due to TLR7-MYD88 hyper-responsiveness, leading to autoimmune-like phenotypes in some individuals (7). This DN axis is mechanistically distinct from haploinsufficiency. Thus, the stimulus (TLR *vs.* TNF) and cellular context (immune cells *vs.* stroma cells) determine the clinical presentation, and knowledge of the mechanism may be useful for selection of different therapies (i.e., when to obtain an IFN-I gene signature and consider a JAK inhibitor) (7).

Although *RELA* variants are most frequently discussed in the context of BD-like or mucocutaneous inflammatory disease, recent evidence indicates that *RELA* plays a broader role in human immune pathology (**Supplementary Table 3**). Thus, pathogenic *RELA* variants are implicated in diverse clinical phenotypes, including autoimmune lymphoproliferative syndrome, type I interferonopathy, and combined immune dysregulation, depending on the type of variant and molecular mechanism. These observations highlight the pleiotropic functions of *RELA* in immune homeostasis and underscore that RAID should be viewed as a spectrum rather than a single clinical entity.

In addition to *RELA*, other studies have suggested that other negative regulators of NF-κB signaling may function in BD and related autoinflammatory phenotypes. Notably, pathogenic variants in *TNFAIP3* (encoding the ubiquitin-editing enzyme A20) can cause haploinsufficiency of A20 (HA20), a monogenic disorder that frequently presents with BD-like manifestations. This underscores the central role of down-regulated NF-κB signaling in the pathogenesis of BD (9).

Most previously described patients with RAID presented during childhood or adolescence, consistent with a monogenic form of immune dysregulation. However, disease onset is not restricted to early life, and several individuals with pathogenic *RELA* variants have been reported to develop BD-like manifestations in late adolescence or adulthood (4). These observations suggest that the onset of RAID can occur in a broad age range, potentially influenced by the type of variant, molecular mechanism, and modifying genetic or environmental factors.

Regarding zygosity, the vast majority of reported patients with *RELA*-associated inflammatory disease harbor heterozygous variants, consistent with an autosomal dominant mode of inheritance. These variants typically act through haploinsufficiency or dominant-negative mechanisms. To date, no studies have convincingly reported homozygous *RELA* variants in patients with BD-like or related inflammatory phenotypes.

In agreement, our Patient 2 and the carrier mother had the same transcriptional defect but very different clinical courses (**Figure 4**). Similarly, our Patient1 had symptoms of the classic BD-like mucosal pathology, but Patient2 had a non-ulcerative, organ-specific phenotype dominated by recurrent fever, uveitis and scleritis, and progressive SNHL. Reports of ALPS-like disease and systemic lupus erythematosus-like autoimmunity further underscore diversity of non-mucosal symptoms in these patients (3–6, 8).

We used LPS to stimulate PBMCs isolated from Patient 2 and his carrier mother, and the results indicated impaired up-regulation of *BCL2A1* and *TRAF1* mRNAs which is consistent with a reduced p65/p-p65 ratio and diminished canonical signals reported in large kindreds. By contrast, IL-6 secretion was markedly greater only in the proband, indicating a different cytokine response despite the presence of the same transcriptional defect. This context-dependent cytokine skewing was previously described in familial cohorts, and is a potential explanation for the proband’s hyper-inflammatory flares and the incomplete penetrance in his mother (5).

Our two cases emphasize the value of *RELA* sequencing for children with unexplained periodic fevers plus organ-specific inflammation (e.g., ear/eye involvement), even in the absence of orogenital ulcers or classic autoantibody profiles. This recommendation aligns with recent reviews that categorized inborn errors of immunity due to alterations of different genes that affect the NF-κB−pathway (*TNFAIP3*, *NFKB1*, *OTULIN*, *NEMO/IKBKG*, and *RELA*) as bridging BD and BD-like inflammatory disorders, and with current guidance on primary immunodeficiencies that can mimic pediatric BD, in which a gene-first approach can expedite mechanism-informed therapy (1, 2).

For patients with mucosal-dominant RAID, colchicine and TNF inhibition are effective (8); for patients with systemic inflammation, drugs that block IL-1 signaling (anakinra, canakinumab, and rilonacept) are effective (5). In settings where pediatric IL-1 receptor antagonists are unavailable or only available off-label (as in mainland China), adalimumab is a rational steroid-sparing alternative. In our Patient2, adalimumab plus a tapering steroid regimen led to control of fever, a decreased level of CRP, and stabilization of the hearing thresholds over a 9 month period, and this patient experienced no infectious complications. These outcomes are consistent with previous reports that used anti-TNF agents in different cohorts of patients with *RELA* variants, and are also consistent with other evidence linking TNF-driven cytotoxicity with *RELA* LoF (3–5, 8).

Our *in vitro* TNF-α challenge of murine fibroblasts that were transfected with the *RELA* variant from Patient1 recapitulated the effect of increased apoptosis. Similarly, prior human and murine studies showed that biallelic *RELA* expression protected stromal cells from TNF-mediated death (3, 4). Our *ex vivo* assays of PBMCs from Patient2 and the carrier mother also demonstrated *RELA* transactivation failure based on measurement of *BCL2A1* and *TRAF1* mRNAs in PBMCs from both individuals, yet IL-6 hyper-secretion was only present in the proband, indicative of a dysregulation that depended on the stimulus and cell-type, as previously reported (5). Taken together, these results connect a *RELA* variant to a disrupted signaling pathway, and suggest the potential application of different biomarkers for longitudinal monitoring of these patients.

An intriguing observation in our study is the marked phenotypic discordance between Patient 2 and his carrier mother, who harbor the same *RELA* frameshift variant yet exhibit divergent inflammatory responses, particularly with respect to production of IL-6. Although this phenomenon is often described as incomplete penetrance, a more mechanistically informative explanation may be autosomal random monoallelic expression (aRMAE) or allele-biased expression, sometimes referred to as a transcriptotype. In this process, individual autosomal genes may be preferentially expressed from one allele in a stable or cell-type-specific manner, a phenomenon known as aRMAE or allele-biased expression (10). Such allele-biased expression is increasingly recognized as a contributor to variable expressivity in monogenic immune disorders (11). In this context, differential usage of the wild-type versus mutant *RELA* allele among immune cell populations could plausibly account for the attenuated IL-6 response in the mother compared with the proband. Although not directly examined in the present study, this hypothesis is biologically plausible and testable. Future studies employing allele-specific expression analyses in sorted leukocyte subsets, at baseline and following inflammatory stimulation, may help clarify how transcriptotype-level regulation shapes disease expression in *RELA*-associated inflammatory diseases.

The differences in our Patient 1 (who had BD-like symptoms), Patient 2 (who had systemic-inflammation and SNHL), and Patient 2’s mother (who was a paucisymptomatic carrier) parallels the intrafamilial variability reported in Irish and US kindreds (e.g., frameshift p.His487Thrfs*7; truncation p.Tyr349LeufsTer13) (5), in which disease severity, organ involvement, and cytokine profiles differed in patients with the same genotype (4, 5). Such variability underscores the need for family-based counseling, a proactive approach that includes measurements of ocular and neurological parameters, and selection of a therapy based on measuring the activity of different pathways (1, 7).

When a pediatric patient presents with periodic fever plus organ-specific inflammation (especially of the ears and eyes) and negative serological results, we suggest that clinicians should consider screening for *RELA* variants and related alterations in NF-κB signaling, even when there are no mucosal ulcers. If agents that block IL-1 are available, this is a reasonable first-line option for systemic phenotypes; otherwise, there is mechanistic evidence to support the use of anti-TNF agents (1, 3–5, 8). Although anti-TNF therapy appeared effective in our patients, particularly in the context of major inflammatory indicators, these observations are based on a study of two patients and should be interpreted with caution. The choice of biologic therapy in *RELA*-associated inflammatory disease is likely influenced by disease phenotype, prior treatment response, and local drug availability, and requires individualized clinical judgment. If there is a DN mutation and a high level of IFN, measurements of IFN-I can be used to guide the use of a JAK inhibitor, although evidence supporting this therapy is still limited (7). Finally, the simple functional assays described herein (e.g., LPS stimulation of PBMCs; qPCR of *BCL2A1* and *TRAF1*; and ELISA of IL−6 and TNF) can confirm disruptions of specific pathways and have potential use as pharmacodynamic markers during follow-up (5).

The limitations of this study are that it was a single-center study with only two cases and the follow-up was rather short. In addition, our functional assessments were limited to *in vitro* and *ex vivo* testing and we did not directly analyze affected tissues or interferon profiles. We were unable to distinguish LoF from DN mechanisms and to evaluate the effects of different genetic and environmental factors. Finally, our treatment choices were limited by local availability, and evidence of potential therapeutic efficacy was inferred from previous case series or observational studies (1, 3–5, 7, 8).

## Conclusions

A frameshift mutation in the TAD of *RELA* can cause heterogeneous pediatric phenotypes, from BD-like ulceration to systemic inflammation with SNHL. Our combined use of structural mapping with functional assays indicated that the transactivation failure of *RELA* can lead to stimulus-dependent cytokine skewing. Characterization of these alterations may aid in selection of the most appropriate therapy.

## Data Availability

The raw data supporting the conclusions of this article will be made available by the authors, without undue reservation.

## References

[1] Perazzio SF Allenspach EJ Eklund KK Varjosalo M Shinohara MM Torgerson TR . Behçet disease (BD) and BD-like clinical phenotypes: NF-κB pathway in mucosal ulcerating diseases. Scandinavian J Immunol. (2020) 92:e12973. doi: 10.1111/sji.12973, PMID: 32889730

[2] Shiraki M Kadowaki S Kadowaki T Kawamoto N Ohnishi H . Primary immunodeficiency disease mimicking pediatric bechet’s disease. Children (Basel Switzerland). (2021) 8. doi: 10.3390/children8020075, PMID: 33499153 PMC7911745

[3] Badran YR Dedeoglu F Leyva Castillo JM Bainter W Ohsumi TK Bousvaros A . Human RELA haploinsufficiency results in autosomal-dominant chronic mucocutaneous ulceration. J Exp Med. (2017) 214:1937–47. doi: 10.1084/jem.20160724, PMID: 28600438 PMC5502421

[4] Adeeb F Dorris ER Morgan NE Lawless D Maqsood A Ng WL . A novel RELA truncating mutation in a familial behçet’s disease-like mucocutaneous ulcerative condition. Arthritis Rheumatol (Hoboken NJ). (2021) 73:490–7. doi: 10.1002/art.41531, PMID: 32969189

[5] Lecerf K Koboldt DC Kuehn HS Jayaraman V Lee K Mihalic Mosher T . Case report and review of the literature: immune dysregulation in a large familial cohort due to a novel pathogenic RELA variant. Rheumatol (Oxford England). (2022) 62:347–59. doi: 10.1093/rheumatology/keac227, PMID: 35412596 PMC9960492

[6] Comrie WA Faruqi AJ Price S Zhang Y Rao VK Su HC . RELA haploinsufficiency in CD4 lymphoproliferative disease with autoimmune cytopenias. J Allergy Clin Immunol. (2018) 141:1507–10.e8. doi: 10.1016/j.jaci.2017.11.036, PMID: 29305315 PMC5889970

[7] Moriya K Nakano T Honda Y Tsumura M Ogishi M Sonoda M . Human RELA dominant-negative mutations underlie type I interferonopathy with autoinflammation and autoimmunity. J Exp Med. (2023) 220. doi: 10.1084/jem.20212276, PMID: 37273177 PMC10242411

[8] An JW Pimpale-Chavan P Stone DL Bandeira M Dedeoglu F Lo J . Case report: Novel variants in RELA associated with familial Behcet’s-like disease. Front Immunol. (2023) 14:1127085. doi: 10.3389/fimmu.2023.1127085, PMID: 36926348 PMC10011480

[9] Zhou Q Wang H Schwartz DM Stoffels M Park YH Zhang Y . Loss-of-function mutations in TNFAIP3 leading to A20 haploinsufficiency cause an early-onset autoinflammatory disease. Nat Genet. (2016) 48:67–73. doi: 10.1038/ng.3459, PMID: 26642243 PMC4777523

[10] Reinius B Sandberg R . Random monoallelic expression of autosomal genes: stochastic transcription and allele-level regulation. Nat Rev Genet. (2015) 16:653–64. doi: 10.1038/nrg3888, PMID: 26442639

[11] Stewart O Gruber C Randolph HE Patel R Ramba M Calzoni E . Monoallelic expression can govern penetrance of inborn errors of immunity. Nature. (2025) 637:1186–97. doi: 10.1038/s41586-024-08346-4, PMID: 39743591 PMC11804961

