# Supplementary material for: Case Report: Diverse pediatric phenotypes of RELA frameshift variants: comparison of two cases
Source: Front Immunol. 2026 Feb 18;17:1756745. doi: 10.3389/fimmu.2026.1756745 (PMC12957222; doi:10.3389/fimmu.2026.1756745)
Supplement: Supplementary file 1 [file Table1.docx]

## Supplementary Table 1. Baseline clinical and laboratory features of Patient 1 (female, 12-year-old) and Patient 2 (male, 10-year-old).

| **Feature** | **Patient 1** | **Patient 2** |
| --- | --- | --- |
| Core presentation | 8‑year recurrent painful oral ulcers; 4‑month genital ulcers; intermittent fever (≤39.5 °C) | 1‑year recurrent high‑grade fever (≤40 °C); progressive bilateral SNHL; no mucosal ulcers; prior uveitis/scleritis |
| Exam at admission | Multiple aphthous scars (gingival/buccal); tender superficial ulcers on labia minora | No oral/genital ulcers; mild conjunctival injection |
| Inflammatory labs (peak) | CRP: 48 mg/L, ESR: 62 mm/h | CRP > 200 mg/L, ESR 97 mm/h |
| Ferritin | Normal | 3500 ng/mL |
| Leukocytes (peak) | 7.30×10^9^/L  (neutrophils: 37.3%) | 23.48×10^9^/L  (neutrophils: 78.6%) |
| Autoimmunity/ HLA | ANA/RF/HLA‑B51: negative | Broad autoantibodies: negative (multiple centers) |
| Imaging | Chest CT: localized thickening of the left pleura; abdominal ultrasound: normal. | Chest CT: normal; abdominal CT: mesenteric lymphadenopathy, decreased hepatic attenuation |
| Audiology/ Ophthalmology | Normal | Moderate-severe bilateral SNHL; history of uveitis/scleritis that was steroid‑responsive |
| Infectious work‑up | Routine screens: unrevealing | Repeated evaluations: unrevealing; *Brucella* repeatedly negative |

Abbreviations: CRP, C-reactive protein; ESR, erythrocyte sedimentation rate; SNHL, sensorineural hearing loss.

Supplementary Table 2. *RELA* variants identified in the two patients and predicted molecular impact

| **Patient** | **Sequencing strategy** | **Inheritance (trio)** | ***RELA* transcript** | **HGVS (cDNA)** | **HGVS (protein)** | **Variant class/region** | **Domain mapping** | **Predicted molecular impact** |
| --- | --- | --- | --- | --- | --- | --- | --- | --- |
| 1 | Trio-based whole-exome sequencing (WES) | *De novo* (parents WT) | NM_021975.4 | c.1392delA | p.Asp465Thrfs*14 | Frameshift (C-terminal truncation) | C-terminal transactivation domain (TAD) | Truncation of the C-terminus with predicted loss of transactivation capacity and impaired canonical NF-κB transcriptional output |
| 2 | Trio-based whole-exome sequencing (WES) | Maternal carrier (paucisymptomatic) | NM_021975.4 | c.1483delG | p.Glu495Serfs*6 | Frameshift (C-terminal truncation) | C-terminal transactivation domain (TAD) | Truncation of the C-terminus with predicted loss of transactivation capacity and impaired canonical NF-κB transcriptional output |

Supplementary Table 3. Published pathogenic *RELA* variants and associated clinical phenotypes

| **Reference** | **RELA variant (cDNA / protein)** | **Domain** | **Mechanism (reported or inferred)** | **Key phenotype(s)** | **Representative treatment(s)** |
| --- | --- | --- | --- | --- | --- |
| Badran et al., 2017 (1) | c.559+1G>A / p.Thr164Profs*12 | RHD | HI | Chronic mucocutaneous ulceration, recurrent fever, gastrointestinal inflammation | Anti-TNF therapy; systemic corticosteroids |
| Comrie et al., 2018 (2) | c.736C>T / p.Arg246* | RHD | HI | ALPS-like immune dysregulation, autoimmune cytopenias, lymphoproliferation | Steroids, immunosuppressants, IVIG |
| Adeeb et al., 2020/2021 (3) | c.1459delC / p.His487Thrfs*7 | TAD | HI (C-terminal truncation) | Familial BD-like mucocutaneous ulceration, rash; intrafamilial variability | Colchicine; anti-TNF agents |
| Lecerf et al., 2022 (4) | c.1044dupC / p.Tyr349Leufs*13 | TAD | HI (C-terminal truncation) | BD-like disease, recurrent oral/genital ulcers, ocular inflammation, IBD-like features | Colchicine; glucocorticoids; anti-TNF |
| An et al., 2023 (5) | c.985C>T / p.Arg329*; c.1153C>T / p.Gln385*; c.1311_1312insA / p.Glu438Argfs*9 | RHD / TAD | HI | Behçet-like syndrome with mucocutaneous ulcers, fever, arthritis; variable penetrance | Colchicine; glucocorticoids; anti-TNF |
| Moriya et al., 2023 (6) | Multiple truncating or splice variants | RHD / TAD | DN | Type I interferonopathy with autoinflammation and autoimmunity | JAK inhibitors; immunosuppression |
| Wang et al., 2025 (review + cases) (7) | c.1166_1184del / p.Gln389fs; c.1416dup / p.Glu473fs | TAD | HI / predicted HI | BD-like disease, systemic autoinflammation; expanded phenotype spectrum | Glucocorticoids; anti-TNF; targeted therapy |
| This study  Patient 1 | c.1392delA / p.Asp465Thrfs*14 | TAD | Predicted HI (C-terminal truncation) | BD-like disease with oral and genital ulcers, recurrent fever | Colchicine; low-dose corticosteroids |
| This study  Patient 2 | c.1483delG / p.Glu495Serfs*6 | TAD | Predicted HI (C-terminal truncation) | Severe systemic inflammation, uveitis/scleritis, progressive SNHL | Glucocorticoids; tacrolimus; tofacitinib; adalimumab |

Abbreviations: RHD, Rel homology domain; TAD, transactivation domain; HI, haploinsufficiency; DN, dominant-negative; BD, Behçetss(male, 10-year-oRELA-associated inflammatory disease; IFN, interferon; ALPS, autoimmune lymphoproliferative syndrome; SNHL, sensorineural hearing loss.

Table notes: Most pathogenic *RELA* variants reported to date are heterozygous, clustering in the C-terminal transactivation domain (TAD) or Rel homology domain (RHD).

C-terminal truncating variants are commonly associated with Behçet disease-like or mucocutaneous-predominant phenotypes, whereas dominant-negative RELA variants more frequently manifest as systemic autoimmunity or interferonopathy, although there is phenotypic overlap. Intrafamilial variability and incomplete penetrance are frequently observed.

**References**

1. Badran YR, Dedeoglu F, Leyva Castillo JM, Bainter W, Ohsumi TK, Bousvaros A*, et al*. Human RELA haploinsufficiency results in autosomal-dominant chronic mucocutaneous ulceration*. The Journal of experimental medicine*. (2017) 214:1937-47. doi:10.1084/jem.20160724.

2. Comrie WA, Faruqi AJ, Price S, Zhang Y, Rao VK, Su HC*, et al*. RELA haploinsufficiency in CD4 lymphoproliferative disease with autoimmune cytopenias*. The Journal of allergy and clinical immunology*. (2018) 141:1507-10.e8. doi:10.1016/j.jaci.2017.11.036.

3. Adeeb F, Dorris ER, Morgan NE, Lawless D, Maqsood A, Ng WL*, et al*. A Novel RELA Truncating Mutation in a Familial Behçet's Disease-like Mucocutaneous Ulcerative Condition*. Arthritis & rheumatology (Hoboken, NJ)*. (2021) 73:490-7. doi:10.1002/art.41531.

4. Lecerf K, Koboldt DC, Kuehn HS, Jayaraman V, Lee K, Mihalic Mosher T*, et al*. Case report and review of the literature: immune dysregulation in a large familial cohort due to a novel pathogenic RELA variant*. Rheumatology (Oxford, England)*. (2022) 62:347-59. doi:10.1093/rheumatology/keac227.

5. An JW, Pimpale-Chavan P, Stone DL, Bandeira M, Dedeoglu F, Lo J*, et al*. Case report: Novel variants in RELA associated with familial Behcet's-like disease*. Frontiers in immunology*. (2023) 14:1127085. doi:10.3389/fimmu.2023.1127085.

6. Moriya K, Nakano T, Honda Y, Tsumura M, Ogishi M, Sonoda M*, et al*. Human RELA dominant-negative mutations underlie type I interferonopathy with autoinflammation and autoimmunity*. The Journal of experimental medicine*. (2023) 220. doi:10.1084/jem.20212276.

7. Wang C, Wang W, Hui X, Hou J, Zhou Q, Li Q*, et al*. Case report and literature review: clinical manifestations and treatment of human RelA deficiency*. Frontiers in immunology*. (2025) 16:1529654. doi:10.3389/fimmu.2025.1529654.
